# Combined Superior Mesenteric Artery and Left Renal Vein Compression With Pelvic Venous Congestion Presenting as Severe Malnutrition in a Young Woman: A Case Presentation

**DOI:** 10.7759/cureus.109631

**Published:** 2026-05-25

**Authors:** Paolo Romero Merino, Christian D Saldaña Beltran, Priscilla Freeman, Jorge Brenis Puican, Daniel Ibanez Vasquez

**Affiliations:** 1 Family Medicine, Hospital Clinico Universitario de Valladolid, Valladolid, ESP; 2 Faculty of Medicine, Universidad Nacional de Trujillo, Trujillo, PER; 3 General Physician, University of Tolima, Colombia, COL; 4 Faculty of Medicine, Catholic University of Santo Toribio de Mogrovejo, Chiclayo, PER; 5 General Physician, Universidad Privada del Valle, Cochabamba, BOL

**Keywords:** aortomesenteric angle, nutcracker syndrome, superior mesenteric artery syndrome, vascular compression, weight loss

## Abstract

Superior mesenteric artery syndrome (SMAS) and Nutcracker syndrome (NCS) are rare vascular disorders, and their simultaneous presentation is exceptional. Both conditions may share a common anatomical basis involving narrowing of the aortomesenteric space, which can compress the third portion of the duodenum and the left renal vein.

We describe a 38-year-old woman who presented with persistent abdominal pain, episodes of nausea, and significant weight loss. Contrast-enhanced CT revealed duodenal obstruction compatible with SMAS and entrapment of the left renal vein suggestive of NCS. Conservative management with nutritional support was initiated, resulting in short-term symptomatic improvement; however, long-term outcomes remain unknown because the patient was transferred to another institution for a planned intervention.

This case highlights the importance of systematically evaluating the aortomesenteric angle and distance in patients with gastrointestinal symptoms following weight loss, as well as assessing the left renal vein to rule out simultaneous compression. Early identification may reduce diagnostic delay and support appropriate management. In patients with substantial weight loss and persistent gastrointestinal symptoms, suspected combined vascular compression should prompt systematic CT-based evaluation of the aortomesenteric angle, aortomesenteric distance, and possible left renal vein compression, followed by multidisciplinary assessment.

## Introduction

Superior mesenteric artery syndrome (SMAS) is a rare clinical entity characterized by compression of the third portion of the duodenum between the abdominal aorta and the superior mesenteric artery, resulting in partial or complete proximal duodenal obstruction. Its pathophysiology is related to the reduction of the aortomesenteric angle and distance, often associated with the loss of retroperitoneal adipose tissue, which normally provides anatomical cushioning. Predisposing factors include significant weight loss, malnutrition, chronic illnesses, and surgical or anatomical conditions that alter the normal aortomesenteric relationship [1].

SMAS is uncommon, with an estimated incidence ranging from 0.013% to 0.3%, and has been reported more frequently in young women, with an approximate female-to-male ratio of 3:2, possibly related to differences in body composition and susceptibility to retroperitoneal fat loss [2].

Clinically, SMAS often presents with nonspecific symptoms such as postprandial abdominal pain, early satiety, nausea, vomiting, and progressive weight loss, which may contribute to a self-reinforcing cycle of reduced intake, malnutrition, and further narrowing of the aortomesenteric space. Due to its low prevalence and nonspecific manifestations, diagnosis is often delayed and requires imaging studies, particularly contrast-enhanced CT, to demonstrate a reduced aortomesenteric angle, decreased aortomesenteric distance, and gastroduodenal dilatation [3].

Additionally, SMAS may coexist with other vascular compression syndromes, such as Nutcracker syndrome (NCS), characterized by compression of the left renal vein between the aorta and the superior mesenteric artery, which may cause hematuria, flank pain, and pelvic venous congestion. The coexistence of both entities is uncommon and represents a diagnostic challenge, especially in patients with chronic abdominal symptoms and weight loss [4].

This report describes a 38-year-old woman with persistent abdominal pain, nausea, and significant weight loss in whom contrast-enhanced CT demonstrated findings consistent with SMAS and concurrent left renal vein compression with pelvic venous congestion.

## Case presentation

A 38-year-old woman was referred from a primary care center for evaluation of progressive unintentional weight loss, generalized weakness, and persistent gastrointestinal symptoms that had not improved with dietary modifications. Her medical history included HPV-related cervical carcinoma in situ treated with conization in 2006. Her obstetric history included two previous vaginal deliveries. No clinically relevant psychiatric history was documented in the clinical record, and no other abdominal or pelvic surgeries were reported. She had no known drug allergies.

Over the previous year, the patient developed progressively reduced oral intake of both solids and liquids, initially associated with postprandial abdominal pain, early satiety, nausea without vomiting, and intermittent mild epigastric pain. She also reported alternating episodes of constipation and diarrhea, without clear precipitating factors. During this period, she first experienced an unintentional weight loss of approximately 6.4 kg from her usual weight of 54 kg, followed by further weight decline. At the primary care evaluation in August 2025, her weight was 45.7 kg and her height was 169 cm, corresponding to a body mass index (BMI) of 16 kg/m² and a total weight loss of 8.3 kg from baseline.

Physical examination revealed a markedly thin woman with evident loss of muscle mass. Laboratory studies did not reveal a clear inflammatory, metabolic, or urinary explanation for her symptoms. The platelet count was 138,000/µL, and urinalysis showed no hematuria. No urinary or pelvic symptoms, including flank pain, gross hematuria, chronic pelvic pain, or pelvic heaviness, were documented.

Contrast-enhanced abdominal CT showed marked gastric dilatation and compression of the third portion of the duodenum between the abdominal aorta and the superior mesenteric artery. The aortomesenteric angle measured approximately 12°, and the aortomesenteric distance was 4 mm, findings compatible with SMAS (Figure 1). No prior abdominal imaging was available for comparison. 

**Figure 1:**
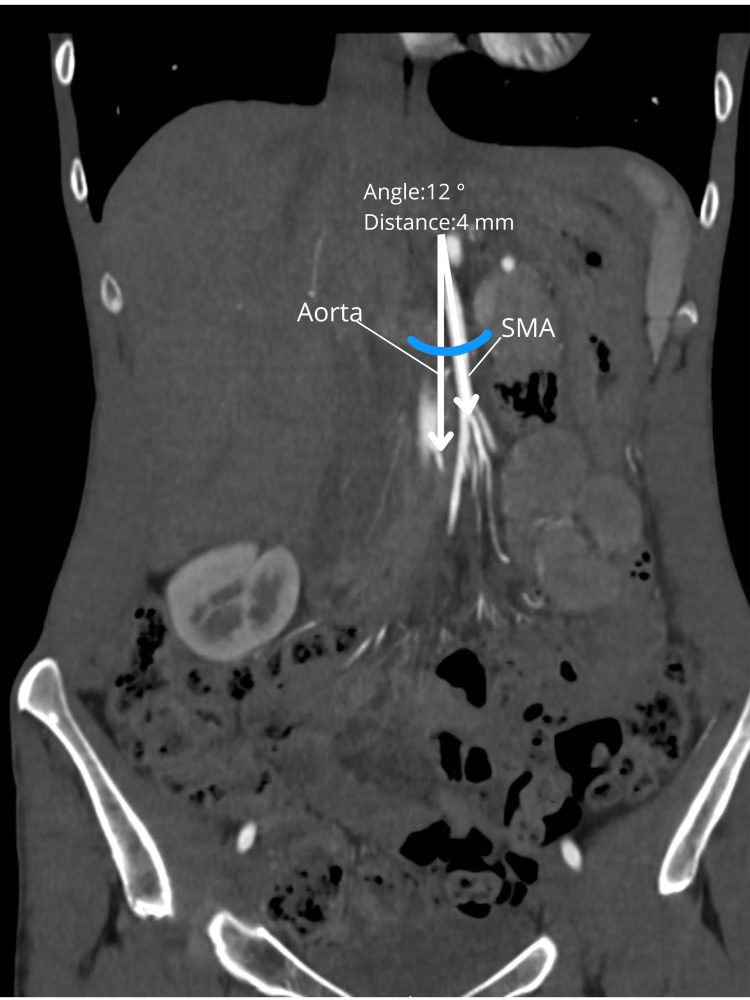
Coronal CT image showing the aorta and SMA with a reduced aortomesenteric angle and a narrowed distance SMA: superior mesenteric artery.

In addition, CT showed narrowing of the left renal vein as it crossed between the aorta and the superior mesenteric artery, consistent with left renal vein compression, or NCS. Associated vascular findings included dilatation of the left ovarian vein and prominent periuterine venous varicosities, compatible with pelvic venous congestion secondary to impaired venous outflow (Figure 2).

**Figure 2:**
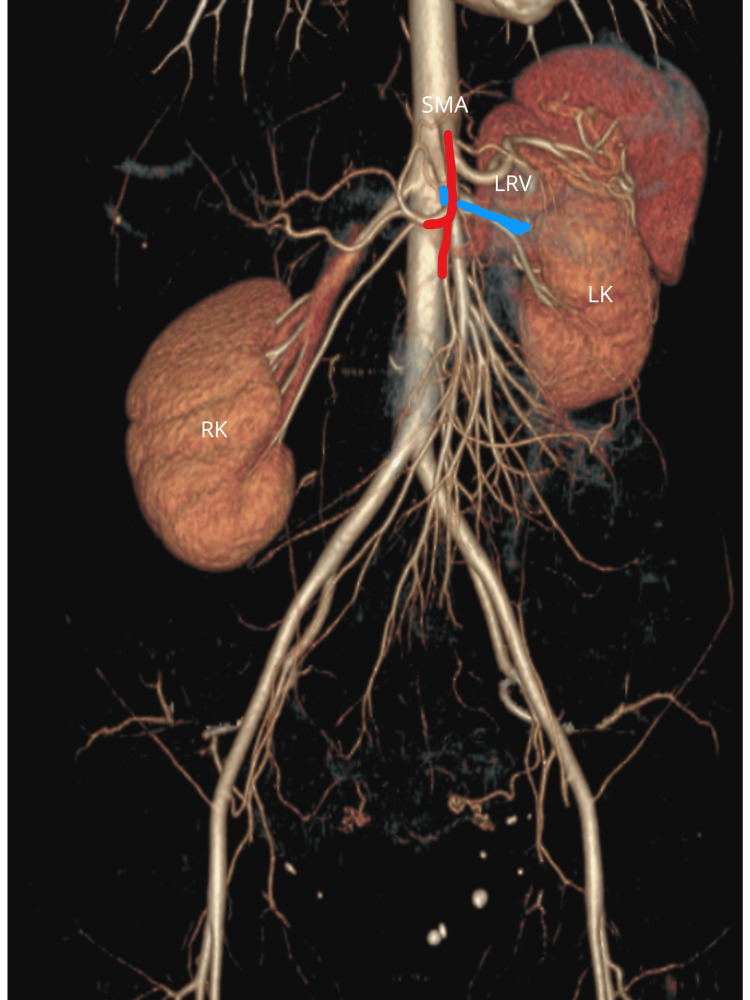
3D abdominal vascular rendering showing the SMA compressing the LRV between the aorta and SMA SMA: superior mesenteric artery, LRV: left renal vein.

The liver, pancreas, and kidneys showed no relevant structural abnormalities. Endoscopic evaluation with duodenal biopsies demonstrated preserved villous architecture, ruling out celiac disease.

Conservative management was initiated with high-calorie nutritional supplementation and dietary modifications. Because of persistent symptoms and the presence of multiple vascular compression findings, the patient was evaluated by the vascular surgery team. During this assessment, the specialists advised an interventional approach targeting the venous component, including coil embolization of the left ovarian vein and stent placement in the left renal vein to address pelvic venous congestion.

The patient was transferred to another institution for the planned intervention; therefore, no surgical or endovascular procedure was performed during the period of care described in this report. No additional imaging, postprocedural information, or long-term follow-up data were available after transfer.

## Discussion

The coexistence of SMAS and NCS is extremely rare, and both conditions are usually diagnosed independently [5]. The incidence of SMAS is low, predominantly in young women. In contrast, the prevalence of NCS is unknown and likely underestimated because of its variable clinical presentation and lack of standardized diagnostic criteria [6,7]. Recent studies report a mean age at presentation of around 28 years, with a slight male predominance. Additionally, previously published case reports show considerable variability in age and sex. Diab et al. described an 18-year-old male [4], Abenavoli et al. reported a 54-year-old woman [5], Khan et al. documented a 25-year-old woman, and Boroomand-Saboor et al. reported a 36-year-old man [8], highlighting that this association can occur across a wide age range without a consistent sex predominance.

Both entities share a common pathophysiology characterized by a critical reduction in the aortomesenteric space [8]. Under normal conditions, retroperitoneal fat acts as a cushion, maintaining adequate separation between the aorta and the superior mesenteric artery. Loss of this adipose tissue leads to decreased angle and distance, promoting compression of both the third portion of the duodenum and the left renal vein [9,10]. Radiologically, SMAS is associated with an aortomesenteric angle of <22-25° and a distance of <8 mm, consistent with this case [6,7].

Traditionally, this condition has been associated with significant weight loss secondary to eating disorders, chronic wasting diseases, or recent surgery [5,6]. In our patient, no clear precipitating factor for weight loss was identified. She had no history of prior abdominal or pelvic surgeries and no relevant gynecologic conditions. She had generally maintained a body weight ranging from normal to slightly below normal until developing a progressive, unintentional 8.3-kg weight loss over the course of her symptoms. In contrast, previously published cases often describe identifiable contributing factors, including surgical history, gastrointestinal pathology, or behavioral triggers. Diab et al. reported a patient with a borderline low BMI and gastrointestinal symptoms associated with weight loss, whose only relevant history was prolonged fasting for religious reasons [4]. Abenavoli et al. described a patient with two gastric polyps, diffuse atrophic gastritis, and prior cholecystectomy, conditions that may contribute to altered digestion and reduced intake [5]. Brogna et al. presented a case involving a patient with a history of mastectomy and three cesarean sections, with the most recent surgery preceding the onset of gastrointestinal symptoms and weight loss, suggesting that postoperative anatomical or functional changes may have played a role [7]. Boroomand-Saboor et al. reported a patient with four weeks of gastrointestinal symptoms and approximately 20 kg of weight loss, also without an identifiable trigger [8].

NCS typically presents with hematuria and left flank pain. However, our patient did not exhibit classic urological manifestations. Instead, she showed signs of pelvic venous congestion, including dilatation of the left ovarian vein and periuterine varicosities. This can be explained by increased venous pressure in the left renal vein, which promotes retrograde flow into the left gonadal vein, resulting in pelvic venous congestion as the predominant manifestation [6,11,12]. Interestingly, previously published cases demonstrate variability in the presence of hematuria. Diab et al. reported a patient without hematuria despite radiologic evidence of left renal vein compression [4]. In contrast, Brogna et al. described a case with microhematuria as part of the clinical presentation [7]. Similarly, Boroomand-Saboor et al. documented a patient without hematuria despite significant vascular compression [8]. These differences highlight that hematuria, although characteristic, is not universally present in NCS and should not be considered a mandatory diagnostic feature.

The overlap between the two entities creates a complex clinical picture that may hinder diagnosis. It is also plausible that the combination of duodenal obstruction and pelvic venous congestion contributed to altered bowel transit, such as alternating diarrhea and constipation, through mechanisms of visceral dysfunction associated with chronic venous congestion [13].

This case broadens the clinical spectrum described in the literature by documenting an atypical presentation of NCS associated with SMAS, in which pelvic venous congestion predominated in the absence of hematuria. Although previous reports have described the coexistence of both entities, most cases presented classic urological symptoms or had identifiable contributing factors such as fasting, gastrointestinal pathology, or prior abdominal surgery. In contrast, our patient exhibited neither hematuria nor a clear precipitating cause for weight loss. This variability highlights the relevance of our case as a rare clinical variant. It underscores that vascular compression syndromes should be considered in patients with unexplained weight loss and persistent gastrointestinal symptoms, even when typical urinary findings or identifiable triggers are absent [14].

Limitations include the absence of specific hemodynamic studies, such as duplex Doppler ultrasonography or venous pressure gradient measurements, which would have allowed more precise characterization of compression severity. Long-term follow-up data are also unavailable to assess clinical evolution after intervention.

Given the rarity of this association, the available evidence is largely based on case reports, underscoring the need for additional studies to establish standardized diagnostic criteria and therapeutic strategies.

## Conclusions

The coexistence of SMAS and NCS represents a rare form of dual vascular compression arising from significant narrowing of the aortomesenteric space. This case illustrates an atypical presentation in which pelvic venous congestion predominated, without hematuria or an identifiable precipitating factor for weight loss. Such variability across reported cases underscores that vascular compression syndromes may occur even in the absence of classic urological symptoms or clear triggers. Clinicians should therefore maintain a high index of suspicion in patients with unexplained weight loss and persistent gastrointestinal symptoms. In these scenarios, systematic evaluation of the aortomesenteric angle and the left renal vein using contrast-enhanced CT with vascular reconstruction is essential to avoid diagnostic delays and guide individualized management.
